# Global Research on Coronaviruses: Metadata-Based Analysis for Public Health Policies

**DOI:** 10.2196/31510

**Published:** 2021-11-30

**Authors:** Thierry Warin

**Affiliations:** 1 HEC Montréal Montréal, QC Canada

**Keywords:** COVID-19, SARS-CoV-2, natural language processing, coronavirus, unstructured data, data science, health 4.0

## Abstract

**Background:**

Within the context of the COVID-19 pandemic, this paper suggests a data science strategy for analyzing global research on coronaviruses. The application of reproducible research principles founded on text-as-data information, open science, the dissemination of scientific data, and easy access to scientific production may aid public health in the fight against the virus.

**Objective:**

The primary goal of this paper was to use global research on coronaviruses to identify critical elements that can help inform public health policy decisions. We present a data science framework to assist policy makers in implementing cutting-edge data science techniques for the purpose of developing evidence-based public health policies.

**Methods:**

We used the EpiBibR (epidemiology-based bibliography for R) package to gain access to coronavirus research documents worldwide (N=121,231) and their associated metadata. To analyze these data, we first employed a theoretical framework to group the findings into three categories: conceptual, intellectual, and social. Second, we mapped the results of our analysis in these three dimensions using machine learning techniques (ie, natural language processing) and social network analysis.

**Results:**

Our findings, firstly, were methodological in nature. They demonstrated the potential for the proposed data science framework to be applied to public health policies. Additionally, our findings indicated that the United States and China were the primary contributors to global coronavirus research during the study period. They also demonstrated that India and Europe were significant contributors, albeit in a secondary position. University collaborations in this domain were strong between the United States, Canada, and the United Kingdom, confirming the country-level findings.

**Conclusions:**

Our findings argue for a data-driven approach to public health policy, particularly when efficient and relevant research is required. Text mining techniques can assist policy makers in calculating evidence-based indices and informing their decision-making process regarding specific actions necessary for effective health responses.

## Introduction

Vaccines against the original SARS-CoV-2 strain have been developed. Public health policies are currently engaged in a battle against new waves of contamination and variants. The political logic is straightforward: the larger the population that has been immunized, the lower the probability of variants. Among their tools, they now have access to new data science tools (eg, machine learning–based analyses and big data, some of which are unstructured) and technological resources, such as high-performance computing platforms. Data science approaches are advantageous, not only for vaccine discovery but also for public health policies.

In this action research–type paper, we use data science techniques to collect and analyze real-time global scientific data. The objective is to examine how data science can be used to improve public health policies. Indeed, with these new tools and data sources, policy makers can (1) conduct the most accurate diagnosis of the current state of knowledge regarding SARS-CoV-2 and (2) act by assisting leading collaborative teams. As a result, decision-making processes at the national and international levels must be optimized. We propose a data science protocol in this paper that could be quickly implemented, for example, with the support of the World Health Organization (WHO), in order to optimize research collaboration across countries, universities, and researchers.

To our knowledge, this is the first paper describing a data science approach for better informing health policy decisions about coronaviruses based on global research.

One of the lessons learned from the SARS-CoV-2 outbreak is the critical nature of public policy responses. Health policy makers must be aware of global research activity. They can, for example, use this information to support some research groups that are closer to developing a vaccine. Another critical feature is that they have real-time access to information, which improves response efficiency. The COVID-19 outbreak exemplifies the critical need for more accurate and timely information. COVID-19 was first identified in late 2019 in Wuhan, China, and some studies were already using data science as a methodology [1]. On January 7, 2020, a novel coronavirus (2019-nCoV) was isolated. Since 2000, two coronavirus outbreaks have occurred: one caused by SARS-CoV and another by the Middle East respiratory syndrome coronavirus (MERS-CoV) [2]. Thus, time is critical.

Another critical factor is having access to the appropriate information. Governments have information about their research groups and their performance based on traditional data collection methods, such as annual reports. However, very few of the world’s close to 200 countries possess this information. Primary sources, on the other hand, are available in the form of research publications. It would first require leveraging all of the metadata contained in these publications. Nowadays, this is possible through the use of natural language processing (NLP) techniques. Second, it would necessitate the development of algorithms to visualize the researchers, countries, and concept networks extracted from these publications. This paper illustrates the use of NLP and social network analysis (SNA) to map the aforementioned networks.

Therefore, our primary contribution is about the utility of a data science–based analysis of global coronavirus research for public health policies. We believe that a detailed map of global research on all coronaviruses is critical. Health care organizations may benefit from such a map. With today’s technologies, this comprehensive mapping can be performed in real time, thanks to a code-based pipeline as illustrated in this paper, allowing for the detection of potential outbreaks of new variants and providing the information necessary to develop subsequent vaccines.

Secondly, a methodological contribution is made. Indeed, we employ metadata in order to conduct an algorithmic review of pertinent literature. In the Methods section, we go into detail about the methodology. It is, in our opinion, a necessary methodological complement to qualitative reviews and meta-analyses.

In short, the primary objective of this paper is to use global research on coronaviruses to identify critical elements that can help inform public health policy decisions. By its very nature, our research question is inscribed in action research. It is methodological and exploratory: in the context of COVID-19 and our technological development stage, how can public health policy makers benefit from machine learning techniques (ie, NLP and SNA) to assist them in their decision making?

## Methods

### Overview

A metadata analysis entails accumulating more articles than a traditional systematic literature review (SLR) and using algorithms to filter and sort the initial data set. We approach this problem in two ways: first, by extracting text-as-data information via NLP techniques, and second, by visualizing potential collaboration networks via SNA.

Combining these two methodological approaches is consistent with Cochrane Reviews’ principle of generating new knowledge through primary research. The primary objective of Cochrane Reviews is to provide information to individuals making health or health care decisions. New research should be designed or commissioned only if it does not duplicate previously conducted research in an unnecessary manner [3]. As a result, an SLR is advantageous prior to initiating any new research, for example, by highlighting specific knowledge gaps or biases [4].

We were inspired by the guidelines for systematic reviews because we used a large data set of research documents. However, our distinction is that our objective was not to contribute to the development of a theoretical framework by identifying distinct research streams (ie, an academic objective) but to propose an example of applied research, more precisely action research.

All of these considerations were particularly pertinent during the COVID-19 period. Thus, the methodology presented in this paper was focused on using the largest data set possible and highlighting some of the mappings that were technologically possible via NLP and SNA.

We formulated two hypotheses about public health policies. First, policies require information about coronavirus research findings. This can assist governments and their various industrial partners in developing pandemic-related solutions. Second, they must be capable of supporting the ecosystems that generate these groundbreaking research findings. During a pandemic—but not exclusively—decision-making processes must be optimized to expedite the production of solutions based on research findings. This means that policy makers must be aware of the characteristics that contribute to the production of these research findings. Individuals (ie, single authors), groups of researchers (ie, multiauthored documents), interuniversity collaborations, or global collaborations are all examples of these characteristics.

**Figure 1:**
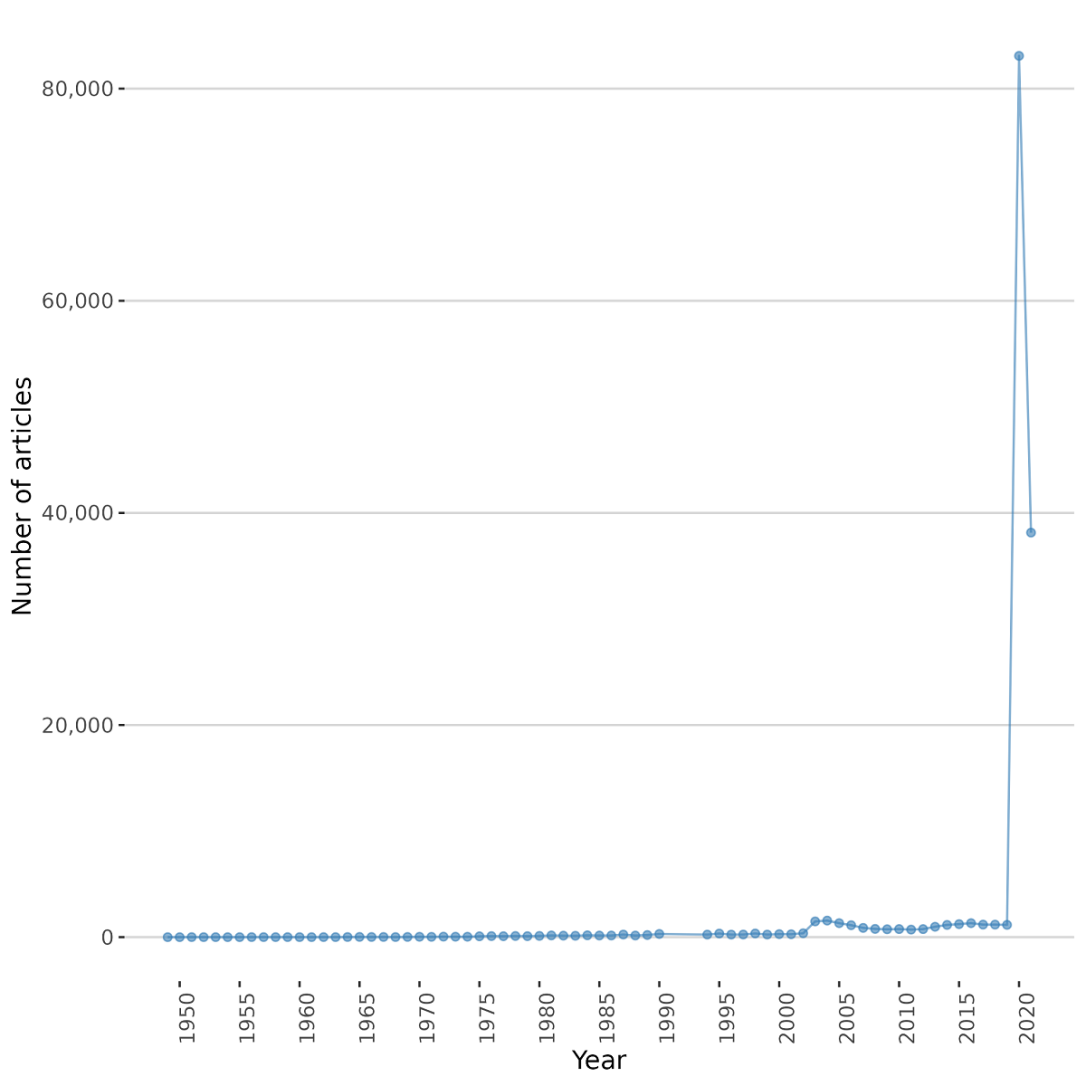
Document count over time. The 2021 document count ended on May 4.

The years 2020 and 2021 logically demonstrate exponential growth in research output (Figure 1).

### Protocol Development

As previously stated, our research question is methodological in nature and exploratory in scope. It is about whether and how public health policy makers can benefit from machine learning techniques to inform their decision-making process in the COVID-19 context and at our technological development stage.

We proposed a four-stage protocol: (1) the first stage required access to global research on coronaviruses, (2) the second stage used NLP techniques to convert the text from published research documents into data, (3) the third stage employed conventional statistical techniques, and (4) the fourth stage used SNA to identify key concepts and collaborators or universities. Interest in SNA has grown in recent years, despite the fact that it is a mathematical field that dates all the way back to the mid-1930s. SNA is predicated on the premise that the social contexts of actions matter [5]. When applied to epidemiology, this means that social contexts matter in coronavirus research, which policy makers should consider.

Each of these four stages would be computer intensive for a researcher but not for a national or international organization. We compiled the algorithms on a dedicated server built with an AMD Ryzen Threadripper processor (Advanced Micro Devices) with 32 cores (64 threads) at 3.2 GHz clock speed, with 128 GB memory.

The first stage involved the collection of data on coronavirus research conducted globally. In the fall of 2019, precisely zero scientists were investigating COVID-19, which was unknown at the time. SARS-CoV-2, the coronavirus that causes the disease, had not yet been identified or named. By the end of March 2020, the disease had spread to over 170 countries and sickened over 750,000 people, and thousands of researchers had shifted their focus away from whatever intellectual challenges had previously piqued their interest and toward the pandemic [6].

In this context, our data collection relied on the EpiBibR (epidemiology-based bibliography for R) package available on GitHub [7]. EpiBibR is a free resource based on open science principles (ie, reproducible research, open data, and open code). The package proposes 22 embedded metadata features and provides access to more than 120,000 references (N=121,231) from July 1, 1949, to May 4, 2021. Being a data package, it provides easy access to the data in order to be integrated efficiently in almost any researcher’s pipeline through the R language [8]. The references were collected via PubMed, a free resource that is developed and maintained by the National Center for Biotechnology Information at the US National Library of Medicine, located at the National Institutes of Health. PubMed includes over 30 million citations from biomedical literature. More specifically, the EpiBibR package adopted the procedure used by the Allen Institute for AI (artificial intelligence) for their COVID-19 Open Research Dataset (CORD-19) project. EpiBibR applies a similar query on PubMed with the following keywords: “COVID-19” OR “coronavirus” OR “corona virus” OR “2019-nCoV” OR “SARS-CoV” OR “MERS-CoV” OR “severe acute respiratory syndrome” OR “Middle East respiratory syndrome” [9]. To the best of our knowledge, the EpiBibR package is the only data package in R providing access to the global research on coronaviruses. This package is updated daily allowing us to build a real-time analysis. It is also the only one of this size. We were able to generate a data set of research documents as of May 4, 2021 (N=121,231). All of these references are accessible through the package [7]. We used the already-available metadata from the package and then, through NLP techniques, we also generated new metadata as explained further below.

For the second and fourth stages, we used the Bibliometrix package in R (version 3.1.4; The R Foundation) on top of our own algorithms, notably to perform disambiguation of authors’ names or to build the SNA [10]. We also created new metadata from the title, the abstract, the keywords, and the references. The latter was particularly computing intensive. Indeed, the algorithm scanned all the references in the references section of each paper. Metadata were generated using NLP techniques. To begin, we prepared the data set by choosing tokens and n-grams [10].

These attributes were required for conducting quantitative analysis on the sample. We were able to create a synthesis of research by using these machine learning tools in conjunction with other techniques, such as SNA. Additionally, the dynamics of research contributions, collaborations, idea generation, and dissemination were examined.

### Study Design

The publishing landscape has shifted due to the introduction of new vehicles and practices, such as preprint servers and open data [11]. Technological advances have also provided access to new methods, such as NLP and machine learning, to complement more conventional SLRs or to present findings when a meta-analysis is not possible [12].

The SLR process is one that enables the collection of pertinent evidence on a given topic that meets predefined eligibility criteria and provides an answer to the formulated research questions. Meta-analyses employ descriptive and/or inferential statistical methods to pool data from multiple studies on a single subject. Thus, the techniques enable knowledge to be generated from a variety of qualitative and quantitative studies. The conventional method entails four basic steps: (1) search (define the search string and database types), (2) appraisal (use predefined criteria for literature inclusion and exclusion, as well as quality-assessment criteria), (3) synthesis (extract and categorize the data), and (4) analysis (narrate the results and, finally, reach a conclusion) [13].

The SLR process is defined as a “systematic, explicit, and reproducible method for identifying, evaluating, and synthesizing the existing body of completed and recorded work” [14]. According to Lasserson et al (page 1) [15], “A systematic review attempts to collate all the empirical evidence that fits pre-specified eligibility criteria in order to answer a specific research question.”

SLRs are not intended to be exhaustive or to be performed in real time. As a result, to complement SLRs, we proposed mapping the entire global research on coronaviruses, given the field’s rapid advancement. The large data set allowed us to analyze the metadata associated with the documents, such as the authors’ affiliations, universities, and references.

Another significant contribution of this new methodology is the computational treatment based on NLP techniques to convert the text to data. As such, NLP in systematic reviews is not new, and some articles have reflected on the interests of NLP techniques [16-18]. In particular, a first set of papers were about information extraction using NLP toolkits like scispaCy [19] or language-based models like BioBERT (bidirectional encoder representations from transformers for biomedical text mining) [20,21]. Another set of papers was about text classification and sentence extraction using BERT [22,23]. Using the CORD-19 data set from the Allen Institute for AI, some other papers have used paper titles and abstracts to build word pairs and co-occurrences to build knowledge graphs highlighting the existence of networks [24,25].

In this paper, we extended these NLP techniques by constructing a series of SNAs using the metadata. We were able to uncover research patterns, research history, and the actual research vehicles, as well as connect discoveries to institutions, to name a few examples. Co-occurrences in the titles and abstracts of each paper were used to highlight the findings from our SNAs.

Finally, another critical dimension was more specific and pertains to the use of each document’s references section. By concentrating on the metrics, researchers can decipher patterns of knowledge transmission. Due to the sheer volume of data being analyzed, this information can only be accessed via an algorithmic approach.

Additionally, we were cognizant of the exploratory nature of our research, employing tools and techniques whose validity had yet to be established. O’Mara-Eves et al [16] documented the biases introduced by machine learning techniques used in systematic reviews. Hopefully, this paper contributes like many others to this healthy and necessary trial and error exercise in terms of scientific validity [17]. Indeed, these new techniques may be used to save time by automating certain tasks, to act as a secondary screener, and to provide new analytical options, such as SNA. This latter point is precisely why this paper exists, particularly in the context of public health policies.

We organized the presentation of the results of these computations using the following theoretical framework. Aria and Cuccurullo [10] suggested examining three distinct structures in their study design—conceptual, intellectual, and social structures—which we did as follows:

1. The conceptual structures were concerned with leveraging the metadata to understand better which concepts and topics are used and how they have evolved in academic discourse.
2. The intellectual structures helped us in determining who originated these concepts, which journals aided in the establishment of this nascent literature, and which articles were most frequently cited in the establishment of this literature.
3. Finally, the social structures enabled us to investigate authors’ collaborations and the knowledge support provided by universities and countries due to those collaborations.

### Data Extraction and Quality Assessment

The relevant “universe” of the literature consists of references from EpiBibR (Table 1)**,** totaling 121,231 documents, most of which have been published in refereed journals (Table 2). The literature review covered the period between January 1, 2020, and May 2021.

The year 2020 has seen an exponential growth of papers on coronaviruses, and 2021 seems to be a replication of 2020. The average citations per document were 0.04 with the information we had. It is a low number, probably explained by the fact that these publications were published in the last few months. As a reference point, the total citations per paper in clinical medicine for the highly cited papers were 5.78 for the 2017-2021 period (Clarivate Analytics, 2021). As seen in Table 1, the documents were published within 7160 different sources, a diverse set of publication vehicles.

Table 2 summarizes the documents’ classifications. The results may be conservative, as some references in the original data set may not contain all of the necessary information. Taking this limitation into account, articles dominated the sample for the entire period (Table 2), accounting for 88,374 occurrences, followed by 16,405 preprints and letters. There have been 120 SLRs published. To summarize, brief contributions (ie, articles and preprints) served as a proxy for the final product.

Consider the metadata generated from the authors’ names and the keywords chosen by the authors of the documents. Coronavirus research on a global scale encompassed 5118 keywords during the overall period (Table 3). It is also worth noting for policy makers that this is a research agenda that interests 377,405 authors. There are a plethora of potential questions raised by these data in the context of public health policy. Additionally, the majority of publications were multiauthored, indicating the increasingly collaborative nature of domain research.

Additionally, the descriptive statistics analysis revealed an average of 3.11 authors and 7.15 coauthors for each publication (Table 4). The vast majority of documents were collaboratively written. Only 13,794 documents were written by a single individual (Table 4 [26]).

Now consider the three distinct structural components: conceptual, intellectual, and social. The first two are required to complete the descriptive statistics aspect.

**Table 1:** Preliminary information about data during the overall period and per year.

Information	Overall time period: 2020-2021	2020	2021
Sources (journals, books, etc), n	7160	6142	4982
Documents, n	121,231	83,090	38,141
Average years from publication	0.685	1	0
Average citations per document	0.04664	0.06746	0.001285
Average citations per year per document	0.02352	0.03373	0.001285

**Table 2:** Document type during the overall period and per year.

Type of document	Overall time period: 2020-2021, n	2020, n	2021, n
Case report	3294	2211	1083
Classical article	2	0	2
Clinical conference	7	5	2
Clinical study	2	2	0
Clinical trial	13	7	6
Clinical trial protocol	41	39	2
Clinical trial, phase II	1	1	0
Comparative study	69	58	11
Congress	8	5	3
Consensus development conference	5	4	1
Editorial	5766	4622	1144
English abstract	1664	1174	490
Equivalence trial	1	0	1
Evaluation study	14	11	3
Guideline	15	15	0
Historical article	22	21	1
Interview	32	27	5
Introductory journal article	6	6	0
Journal article	88,374	58,601	29,773
Lecture	2	2	0
Preprint or letter	16,405	13,068	3337
Meta-analysis	9	5	4
Published erratum	492	270	222
Retraction of publication	15	7	8
Review	1	1	0
Systematic review	120	65	55

**Table 3:** Document content and authors during the overall period and per year.

Document content	Overall time period: 2020-2021, n	2020, n	2021, n
Authors’ keywords	5118	4699	2044
Authors	377,405	266,579	188,900
Author appearances	866,589	569,924	296,665
Authors of single-authored documents	8819	6835	2580
Authors of multiauthored documents	368,586	259,744	186,320

**Table 4:** Details about authors’ collaborations.

Collaboration measure	Overall time period: 2020-2021	2020	2021
Single-authored documents, n	13,794	10,324	3470
Documents per author, n	0.321	0.312	0.202
Authors-per-document index^a^	3.11	3.21	4.95
Coauthors per document, n	7.15	6.86	7.78
Collaboration index^b^	3.43	3.57	5.37

^a^The authors-per-document index was calculated by dividing the total number of authors by the total number of articles.

^b^The collaboration index was calculated by multiplying the total number of authors on multiauthored documents by the total number of multiauthored documents [26].

## Results

### Overview

As mentioned in the Methods section, we used Aria and Cuccurullo’s [10] theoretical framework to present our findings. We present, respectively, the conceptual, intellectual, and social structures. For each structure, we present the relevant metrics that are available.

Additionally, as a proof of concept, we generated the necessary metadata and metrics based on the 121,231 total documents. We would encourage future researchers to filter the data set to address their own research questions, for example, by limiting their search to randomized controlled trial documents or even by content, such as proteins. Due to the fact that text is data, a new set of options becomes available.

### Conceptual Structures of the Global Research on Coronaviruses

#### Overview

In the following subsections, we examined the conceptual structures of our sample by analyzing the keywords, their co-occurrences, and the evolution of the topics using a topic modeling technique. To create this conceptual framework, we created a matrix of the keywords and titles of the 121,231 documents.

#### Keyword-Based Metrics

**Figure 2:**
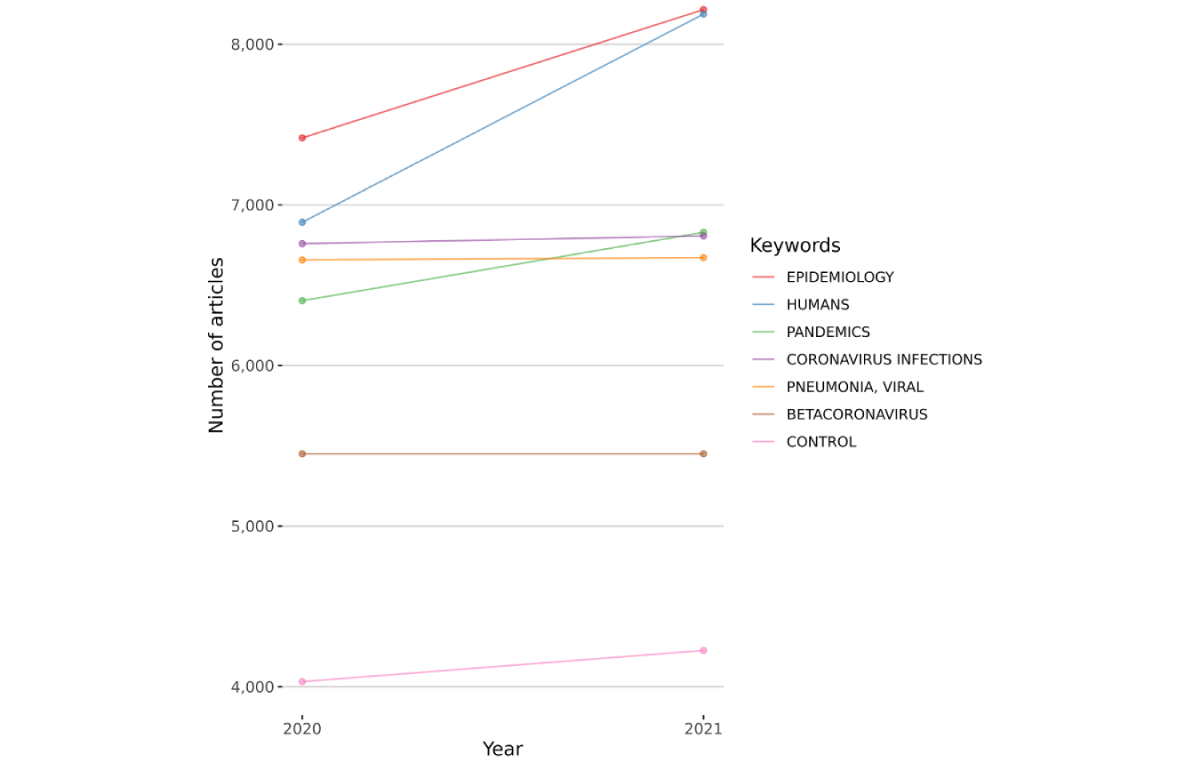
Evolution of the usage of authors’ keywords.

The keyword section of Figure 2 highlights the most frequently used keywords by authors in their documents. Between 2020 and 2021, it was largely stable. Table 5 displays the top keywords in the overall sample and per year.

**Table 5:** Most relevant keywords during the overall period and per year.

Author keywords			Articles where keywords appear (N=121,231), n (%)
**Overall time period: 2020-2021**			
	Epidemiology	8216 (6.8)	
	Humans	8188 (6.8)	
	Pandemics	6829 (5.6)	
	Coronavirus infections	6807 (5.6)	
	Pneumonia viral	6672 (5.5)	
**2021**			
	Humans	1296 (1.1)	
	COVID-19	1246 (1.1)	
	SARS-CoV-2	857 (0.1)	
	Epidemiology	799 (0.1)	
	Pandemics	425 (0.1)	
**2020**			
	Epidemiology	7417 (6.1)	
	Humans	6892 (5.7)	
	Coronavirus infections	6759 (5.6)	
	Pneumonia viral	6658 (5.5)	
	Pandemics	6404 (5.3)	

#### Topic Modeling–Based Analyses Using Keywords

**Figure 3:**
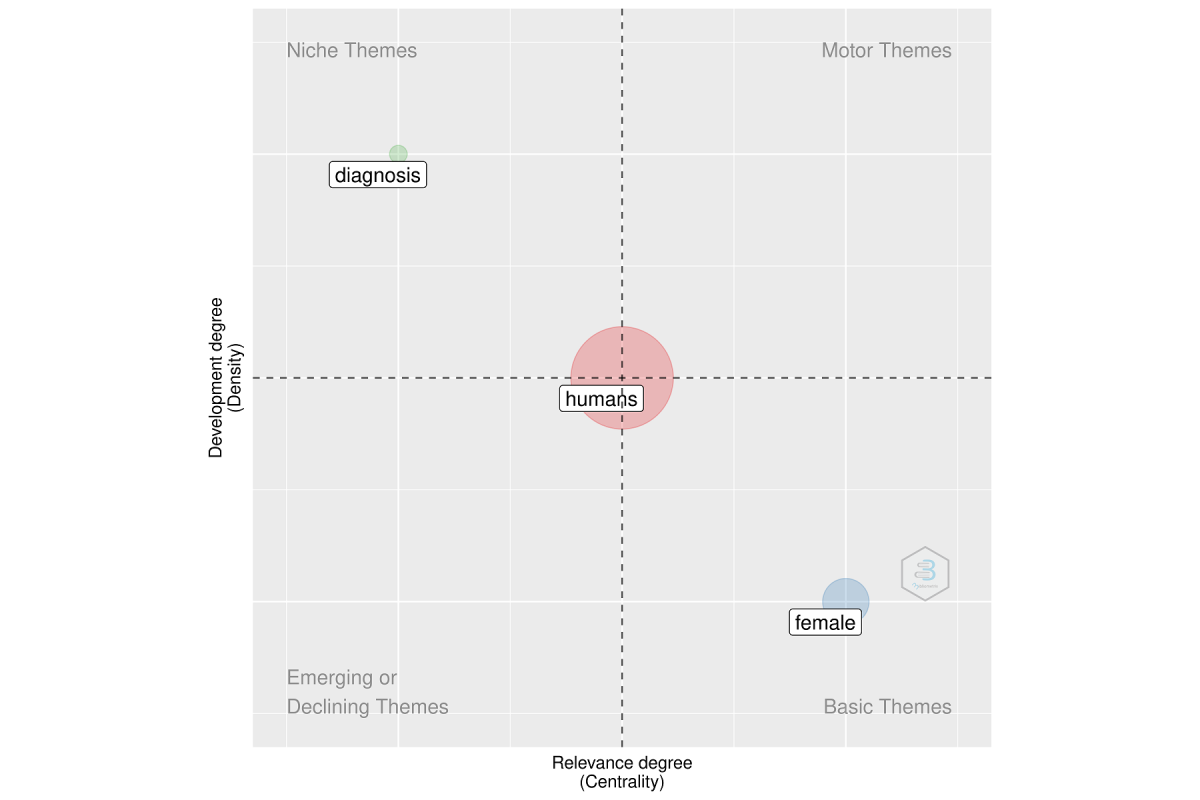
Topic modeling for the overall period.

**Figure 4:**
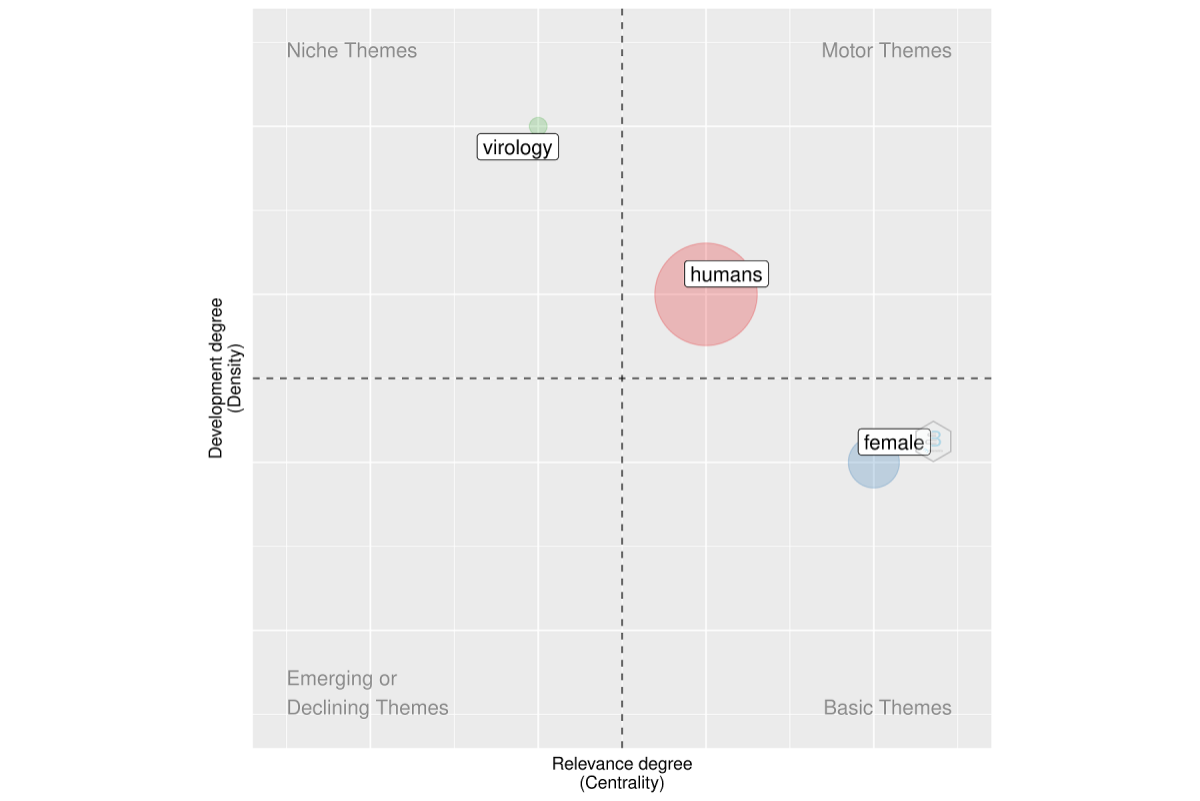
Topic modeling for 2020.

**Figure 5:**
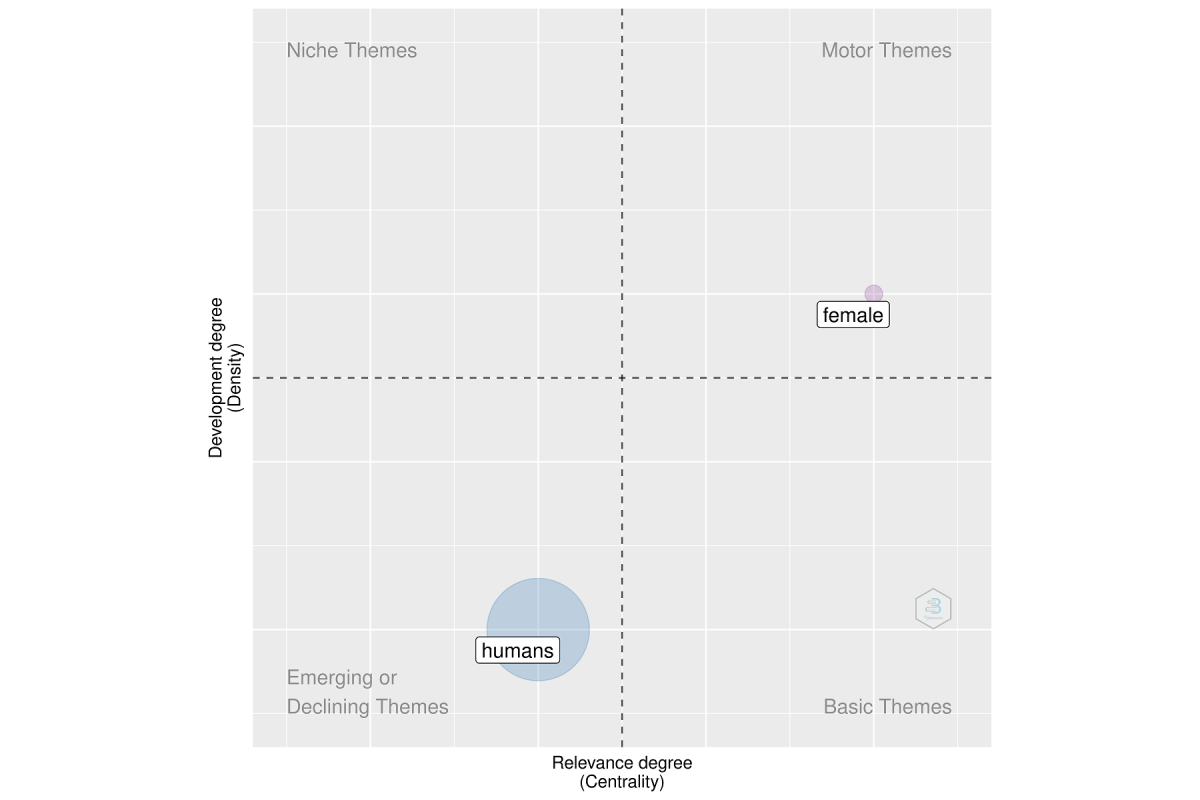
Topic modeling for 2021.

We added a new dimension to the analysis in the following section using structural topic modeling. The purpose of this section is to supplement the information gleaned from keyword co-occurrences. We illustrate this analysis in Figure 3 (overall period), Figure 4 (2020), and Figure 5 (2021). We discovered that the topics were classified into four categories: fundamental themes, emerging or declining themes, niche themes, and motor themes. The results in this case were based on the keywords solely to demonstrate the framework.

The analysis can be carried out using techniques for dimensionality reduction. The following sections make use of multiple correspondence analysis.

We augmented our field’s conceptual structure with k-means clustering in order to identify clusters of documents expressing common concepts solely based on keywords. We used NLP to extract terms from the keywords section. In addition, the algorithm implemented the Porter stemming algorithm to reduce inflected, or sometimes derived, words to their word stem, base, or root form. Finally, we tokenized all the words, and we computed the latent variables to identify potential topics. Because of the necessary high computing power, we performed this analysis on the 2021 data set.

**Figure 6:**
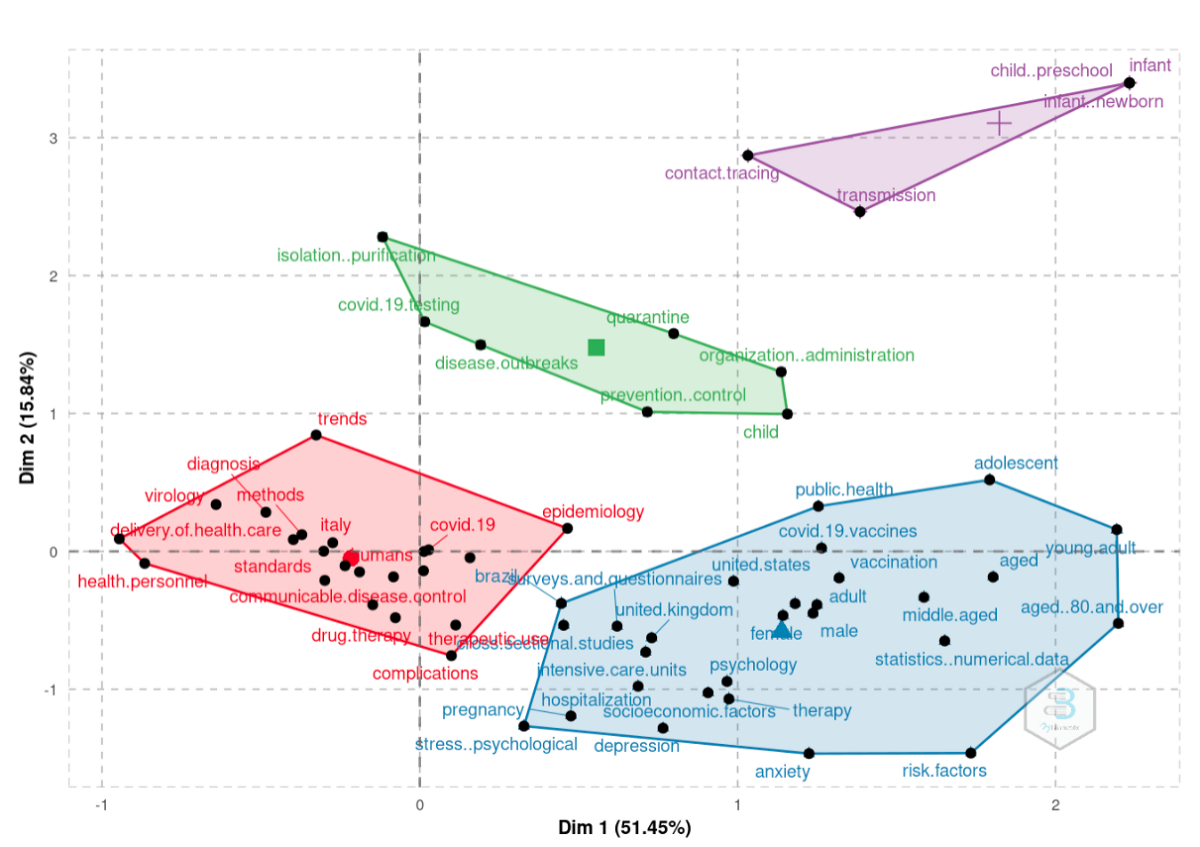
Conceptual structure map based on multiple correspondence analysis. Dim: dimension.

**Figure 7:**
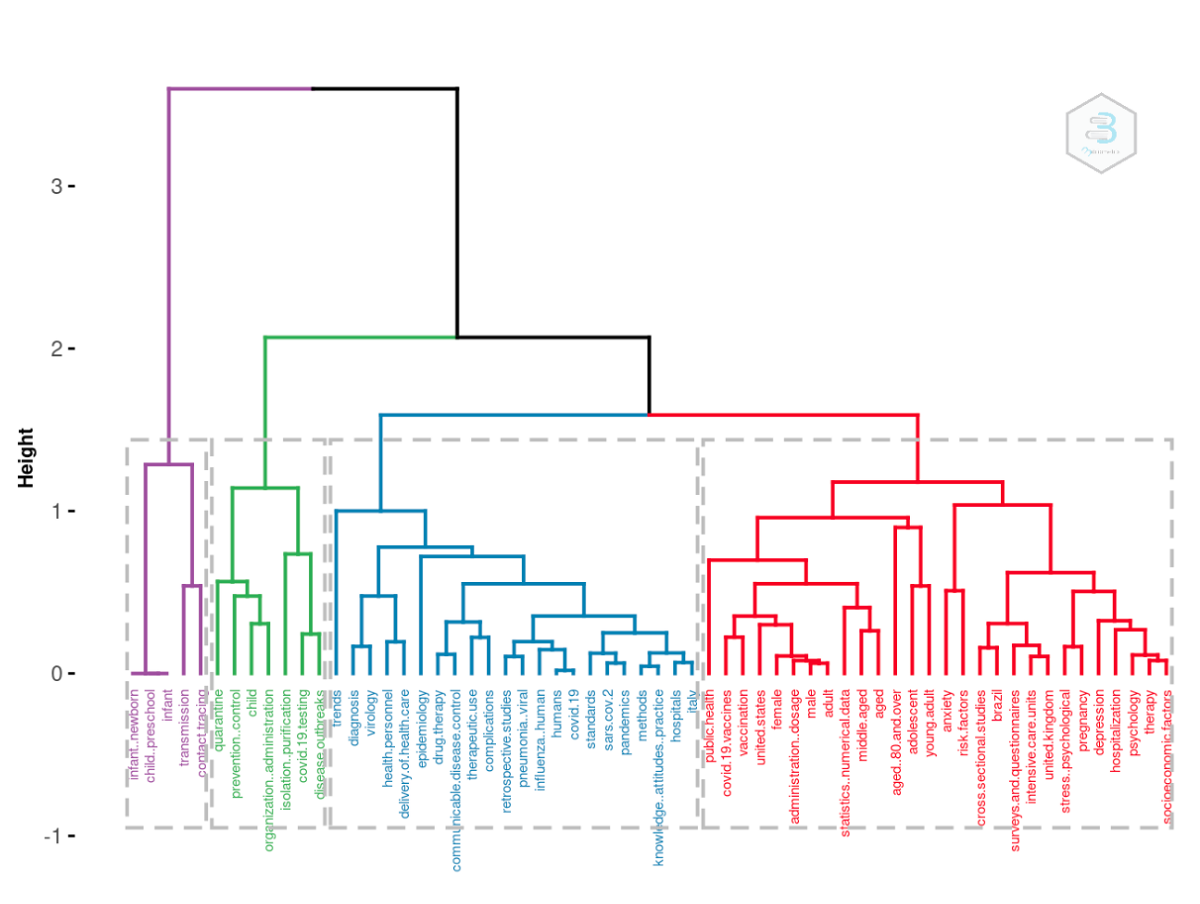
Topic dendogram.

Figures 6 and 7 illustrate ample room for policy implications regarding social distancing and vaccination, respectively (red). The significant topic is population (ie, health status, age, and so on), which is depicted in blue in Figure 6 and red in Figure 7. The same analysis can be performed on additional terms, such as those found in titles, abstracts, or references. As a result, a plethora of potential classifications becomes available.

Following our examination of possible measures of conceptual structures, let us turn our attention to the analysis of intellectual structures.

### Intellectual Structures of the Global Research on Coronaviruses

Another dimension leading to another interesting analysis is to know who, what journals, and which organizations are leaders in these topic dynamics.

#### Author-Based Metrics

In the intellectual structure, authors are interesting to consider for public policies. These metrics come with many biases, as some family names can be prevalent. An important dimension is equity, diversity, and inclusion (EDI). It is not the focus of this paper on public health policy. However, it is possible for future research to delve deeper into this author component of the intellectual structure. With this algorithmic approach and the available metadata, scholars can design EDI metrics to assess, for instance, gender-related questions, such as first and last authors; leadership positions in academia; among others [27-32]. An EDI-based analysis could also correct for the fact that fewer articles have females as the last author and these articles accrue fewer citations per publication [33]. With this metadata-based approach, scholars have access to these metrics. This is a subject that would require a more comprehensive examination of the field as a whole, which is beyond the scope of this work.

**Figure 8:**
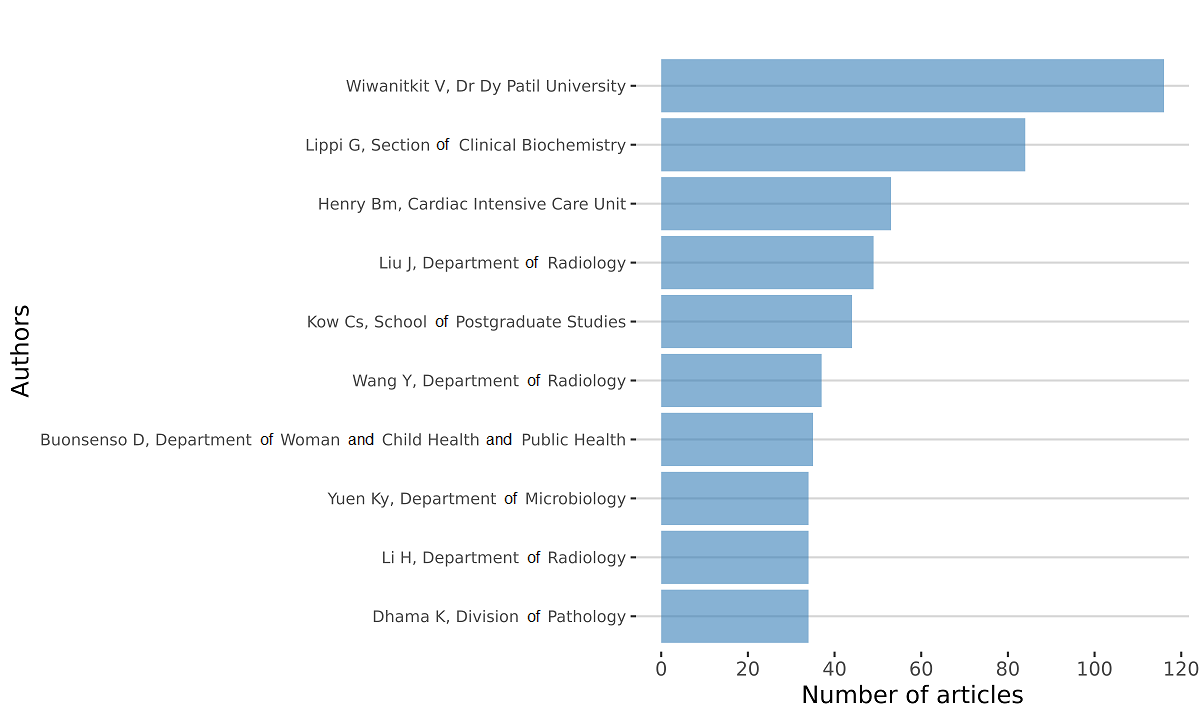
Top authors in terms of production during the overall period.

**Figure 9:**
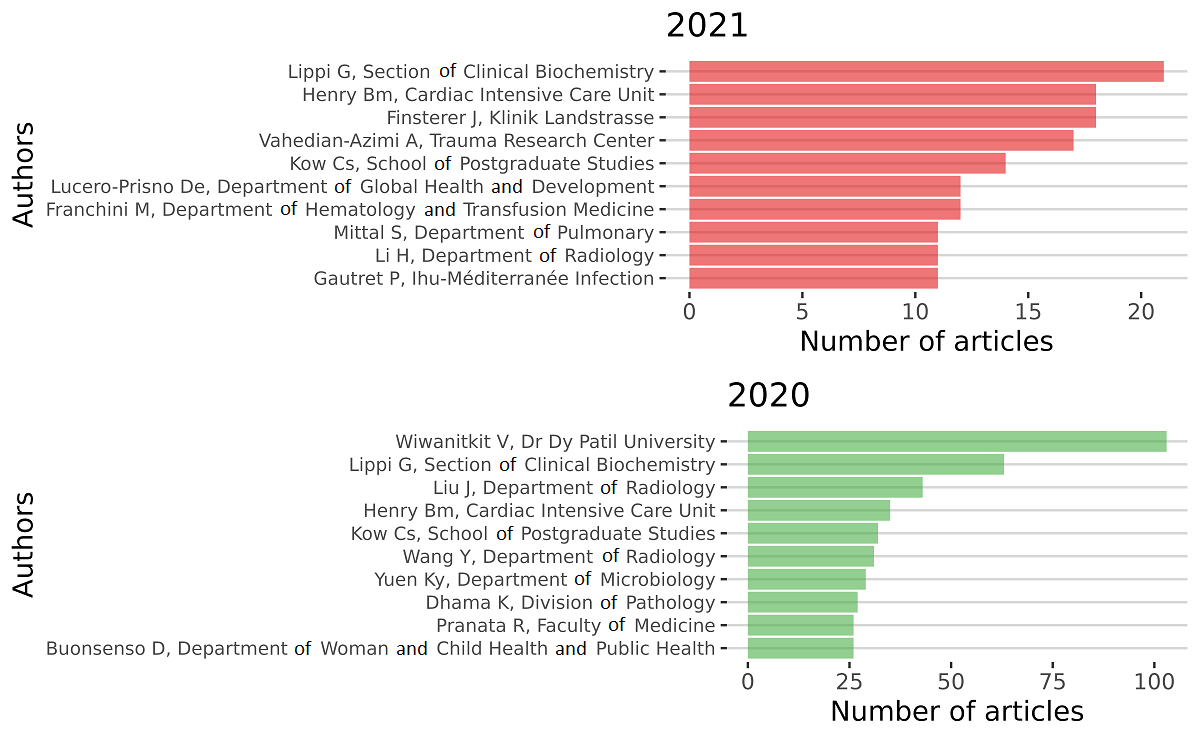
Top authors in terms of production per year.

In Figures 8 and 9, respectively, we present the total count per name for the overall period and per year. It is important to note that homonymy is always an issue to correct. To correct for homonymy, several strategies exist. We could use the ORCID (Open Researcher and Contributor ID) numbers or any other unique identifier. Unfortunately, this information was not available in the original data set. Thus, we designed an algorithm that would associate an author’s name with a university’s name. We sorted the whole data set making sure there were unique pairs of authors and affiliations. Sometimes, university affiliations were written in different forms. We corrected them by creating a dictionary of affiliations to standardize the format.

**Figure 10:**
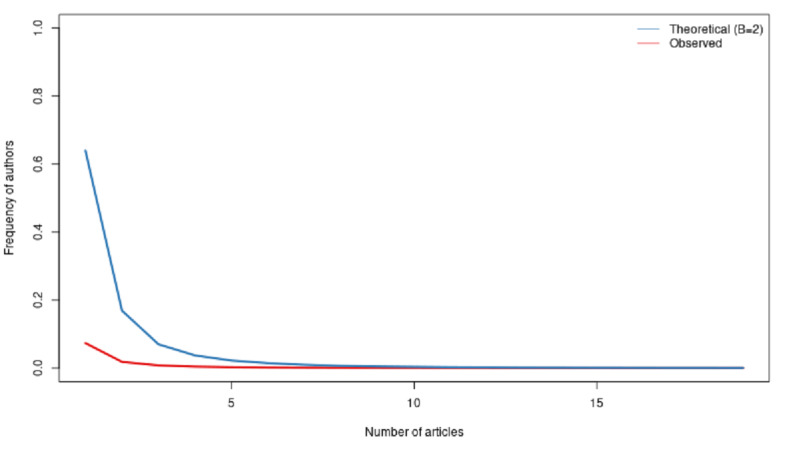
Scientific productivity during the overall period.

**Figure 11:**
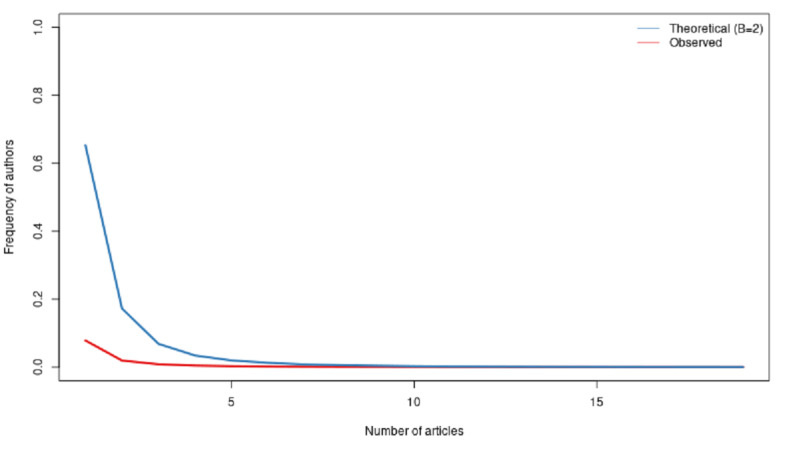
Scientific productivity during 2020.

**Figure 12:**
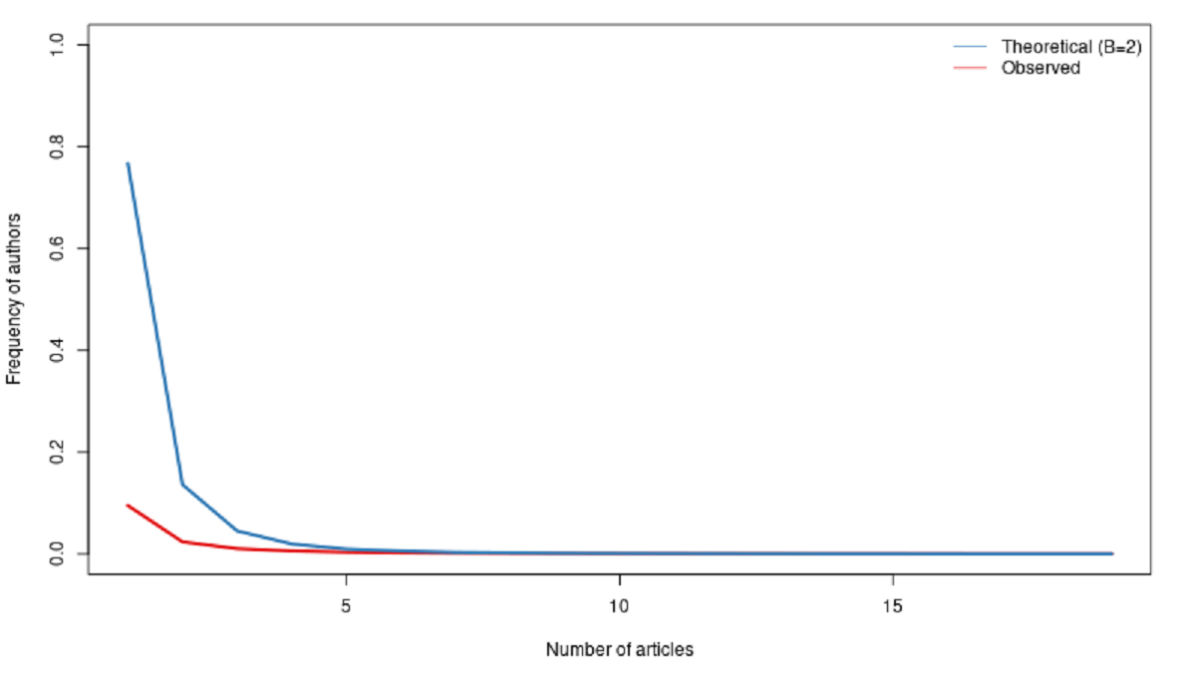
Scientific productivity during 2021.

We can go a little deeper and look at the average productivity of all the authors. One way to design better metrics would be to consider how many articles an author produces per year in our 2-year sample. In Figures 10-12, we computed the Lotka coefficient for the overall period, 2020, and 2021, respectively, to compare the scientific productivity of researchers to the Lotka theoretical coefficient [34]. The Lotka law describes the frequency of publication by authors as an inverse square law, where the number of authors publishing a certain number of articles is a fixed ratio to the number of authors publishing a single article. This assumption implies that the theoretical β coefficient of the Lotka law is equal to 2.

Figures 10-12 describe the share of authors having published a certain number of articles. Here, there was a statistically significant difference between the observed and the theoretical Lotka distributions, meaning that authors were more prolific in this research topic. This does not come as a surprise, considering the urgency of the topic.

Due to the large size of the data set, our dedicated server was not powerful enough to compute the results. Our strategy was, thus, to extract a random sample for 2020 and 2021 of 25,000 documents each year. The 2021 sample corresponded to 65.5% of the total 2021 data set. The 2020 sample corresponded to 30.0% of the total 2020 data set.

To go further, we narrowed it down to specific groups of authors, institutions, or research teams and computed the scientific productivity. It may be relevant, indeed, to allocate resources, as a policy maker, to some of these dimensions.

**Figure 13:**
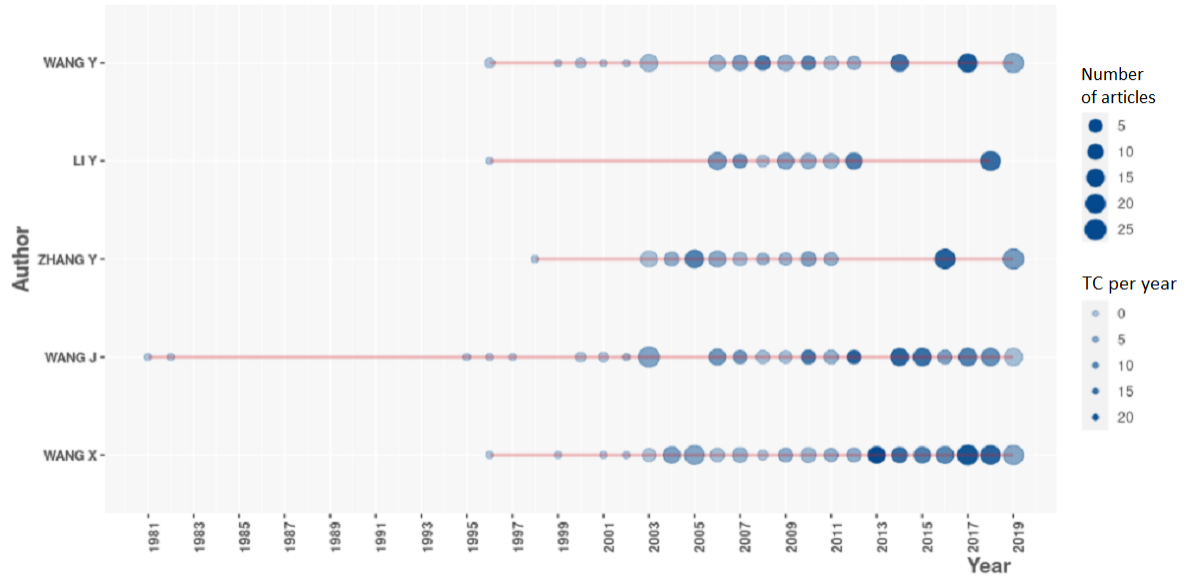
Productivity of the top authors over time. TC: total citations.

To conclude, in Figure 13, we first filtered the original authors’ list to authors having published fewer than 25 articles and to those who had fewer than 20 total citations per year. It was an arbitrary choice, and we could easily filter it differently, which is precisely in line with our main point: data science allows this agile adaptation.

Let us now move to the article element as another interesting dimension to measure intellectual structures.

#### Article-Based Metrics

We had a look at the citations from the data set (N=121,231). Authors represented interesting information regarding public health policies, including their productivity metrics, but we also found it interesting that the most cited manuscripts may help refine the metrics (Table 6).

Let us now go deeper and consider the social structures of the global research on coronaviruses.

**Table 6:** Most cited manuscripts.

Articles (author, year, journal)	Total citations, n	Total citations per year, n
Huang C, 2020, The Lancet	146	73.0
Zhu N, 2020, New England Journal of Medicine	102	51.0
Chen N, 2020, The Lancet	100	50.0
Li Q, 2020, New England Journal of Medicine	89	44.5
Chan JF, 2020, The Lancet	75	37.5
Veljkovic V, 2021, F1000Research	7	7.0
Endo A, 2021, Wellcome Open Research	6	6.0
Wang L, 2021, medRxiv	2	2.0
Fu L, 2021, Clinical Cardiology	1	1.0
Ackermann M, 2021, New England Journal of Medicine	1	1.0

### Social Structures of the Global Research on Coronaviruses

In this section, we focus on different measures to capture the social connections: the co-citations of authors, the co-citations of articles, the co-citations of journals, and the collaborations across institutions.

#### Authors’ Collaboration Metrics

**Figure 14:**
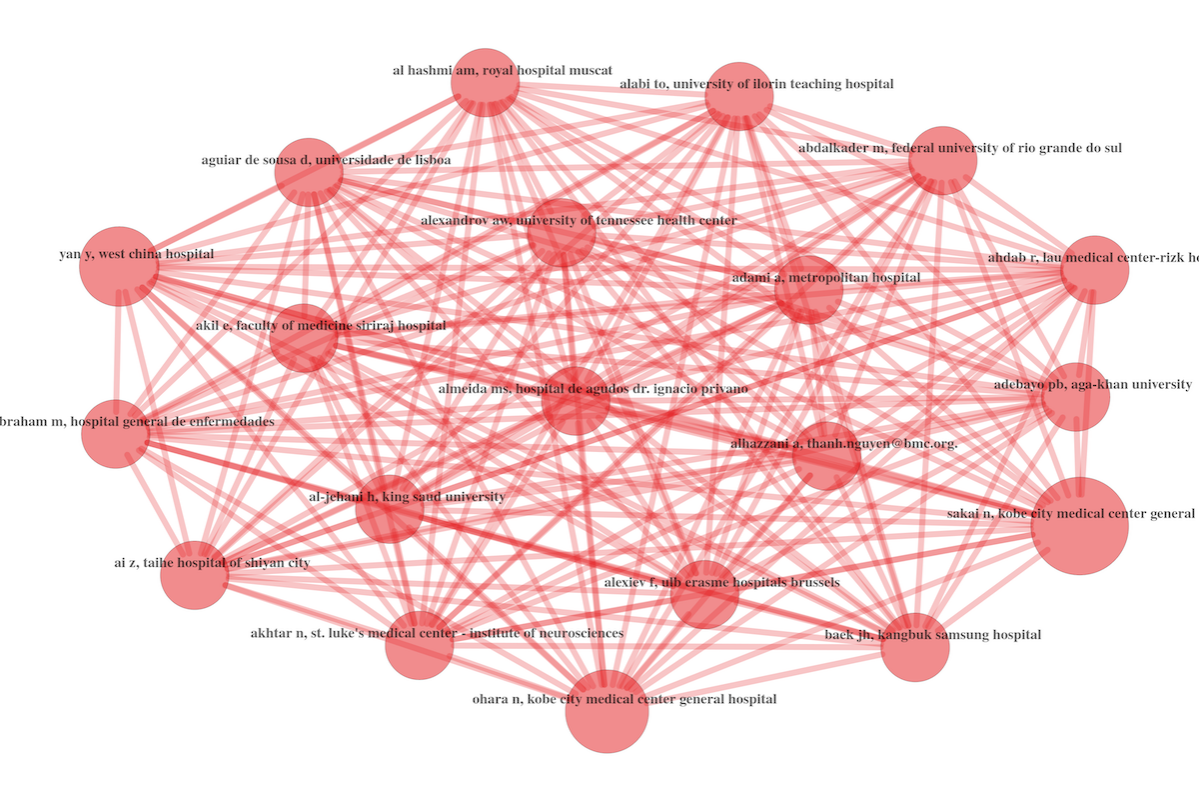
Authors’ collaboration networks in 2021.

Figure 14 highlights the authors’ collaborations. This figure shows the network of the top authors. Again, we can see a high level of collaboration and knowledge transfer. In further research, scholars could also perform the analyses with EDI in mind and use the metadata to have a metric of potential EDI metric imbalances [35]. This can be particularly useful in order to correct these imbalances.

Let us now move our discussion to the country level.

#### Country-Based Metrics

It is also possible to extract country information from the documents. We mapped the top five countries per period. Most of the authors were residents of the United States, the People’s Republic of China, India, and Europe (Table 7).

Table 8 provides supplementary information on the total citations per country. Again, the United States and China dominated the ranking.

**Figure 15:**
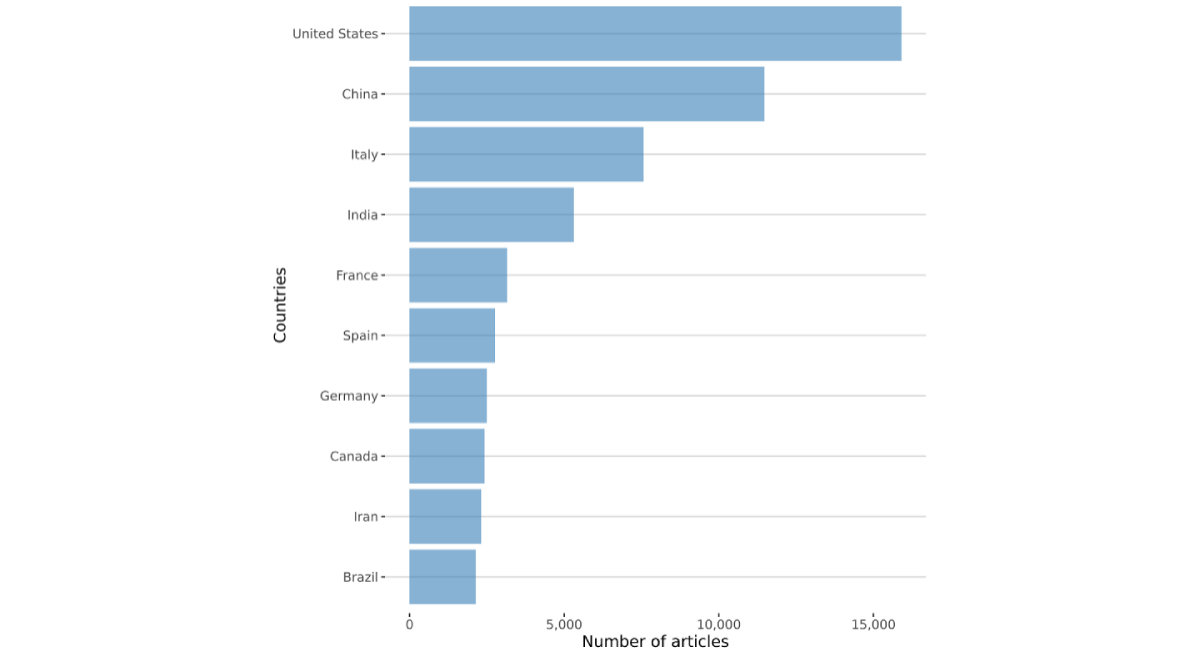
The most productive countries during the overall period, according to authors’ residences.

**Figure 16:**
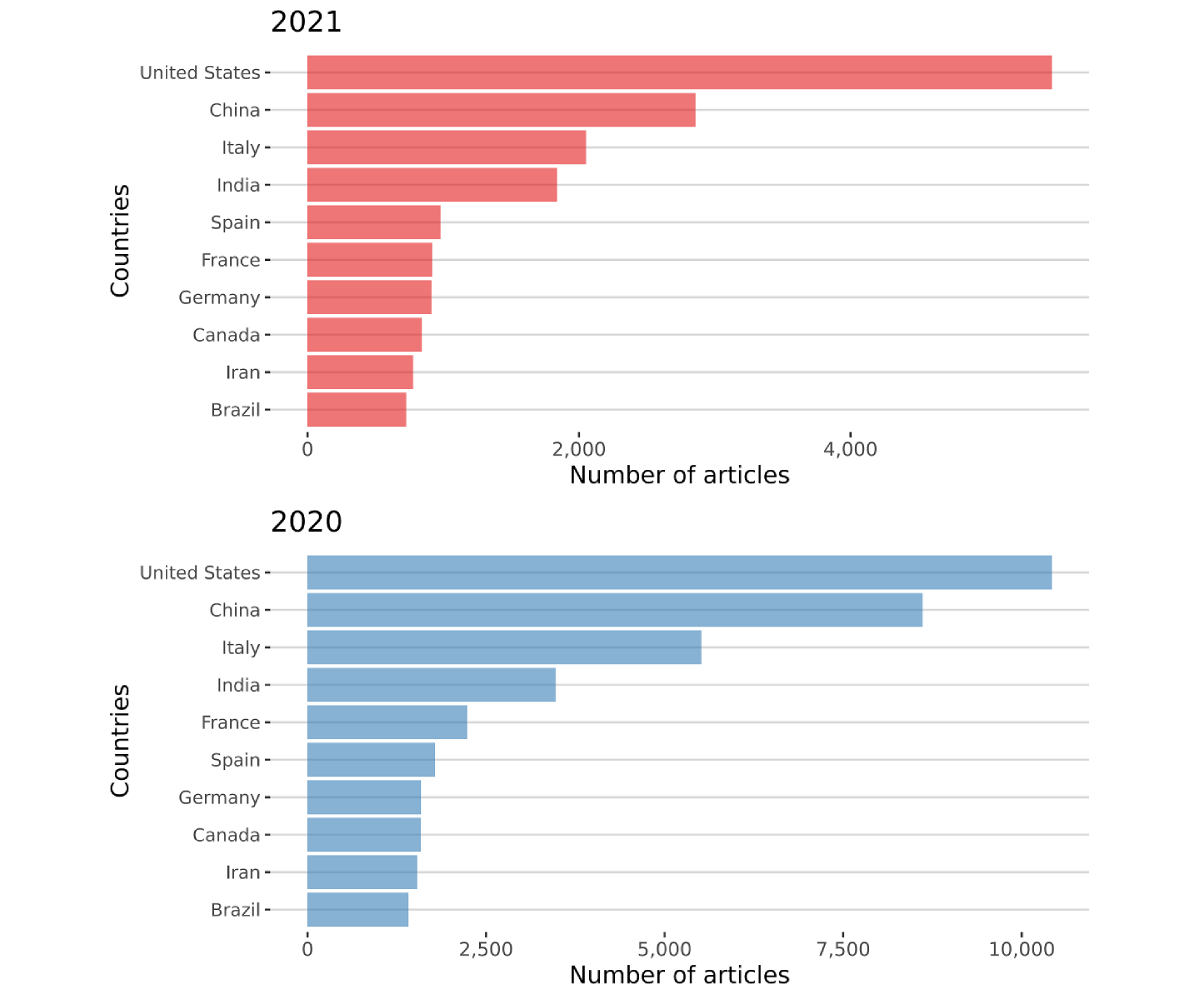
The most productive countries during 2021 (top) and 2020 (bottom), according to authors’ residences.

Figures 15 and 16 show an apparent increase in the contributions coming from Asia: China and India were at the forefront of academic production. Starting from a bibliographic matrix, two groups of descriptive measures were computed: (1) the summary statistics of the network and (2) the leading indices of centrality and prestige of vertices.

**Table 7:** Corresponding authors’ countries during the overall period and per year.

Country		Articles (N=121,231), n (%)	Frequency	Single-country publications	Multiple-country publications	Multiple-country publications ratio
**Overall time period: 2020-2021**						
	United States	15,904 (13.1)	0.1923	15,840	64	0.004024
	China	11,471 (9.5)	0.1387	11,451	20	0.001744
	Italy	7565 (6.2)	0.0915	7533	32	0.004230
	India	5314 (4.4)	0.0643	5295	19	0.003575
	France	3156 (2.6)	0.0382	3139	17	0.005387
**2021**						
	United States	5483 (4.5)	0.2025	5433	50	0.00912
	China	2859 (2.4)	0.1056	2843	16	0.00560
	Italy	2052 (1.7)	0.0758	2022	30	0.01462
	India	1838 (1.5)	0.0679	1824	14	0.00762
	Spain	980 (0.1)	0.0362	975	5	0.00510
**2020**						
	United States	10,421 (8.6)	0.1874	10,407	14	0.001343
	China	8612 (7.1)	0.1549	8608	4	0.000464
	Italy	5513 (4.5)	0.0991	5511	2	0.000363
	India	3476 (2.9)	0.0625	3471	5	0.001438
	France	2237 (1.8)	0.0402	2236	1	0.000447

**Table 8:** Total citations per country during the overall period and per year.

Country		Total citations, n		Average article citation
**Overall time period: 2020-2021**				
	China	2011	0.17531	
	United States	550	0.03458	
	Italy	315	0.04164	
	Germany	131	0.05240	
	France	129	0.04087	
**2021**				
	United States	10	0.001824	
	China	4	0.001399	
	Germany	4	0.004381	
	Belgium	1	0.004484	
	France	1	0.001088	
**2020**				
	China	2007	0.23305	
	United States	540	0.05182	
	Italy	314	0.05696	
	France	128	0.05722	
	Germany	127	0.08003	

**Figure 17:**
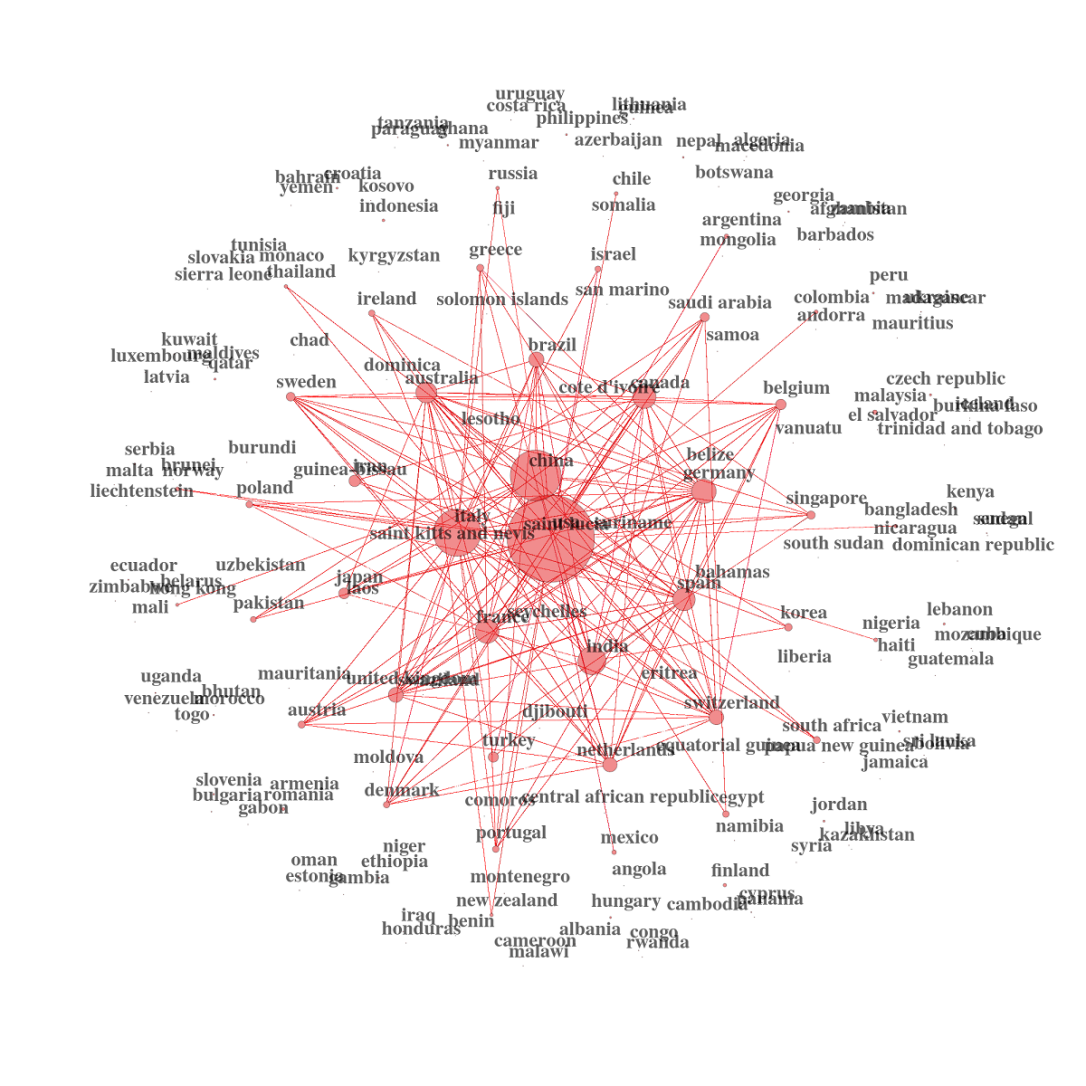
Country collaboration networks during the overall period.

**Figure 18:**
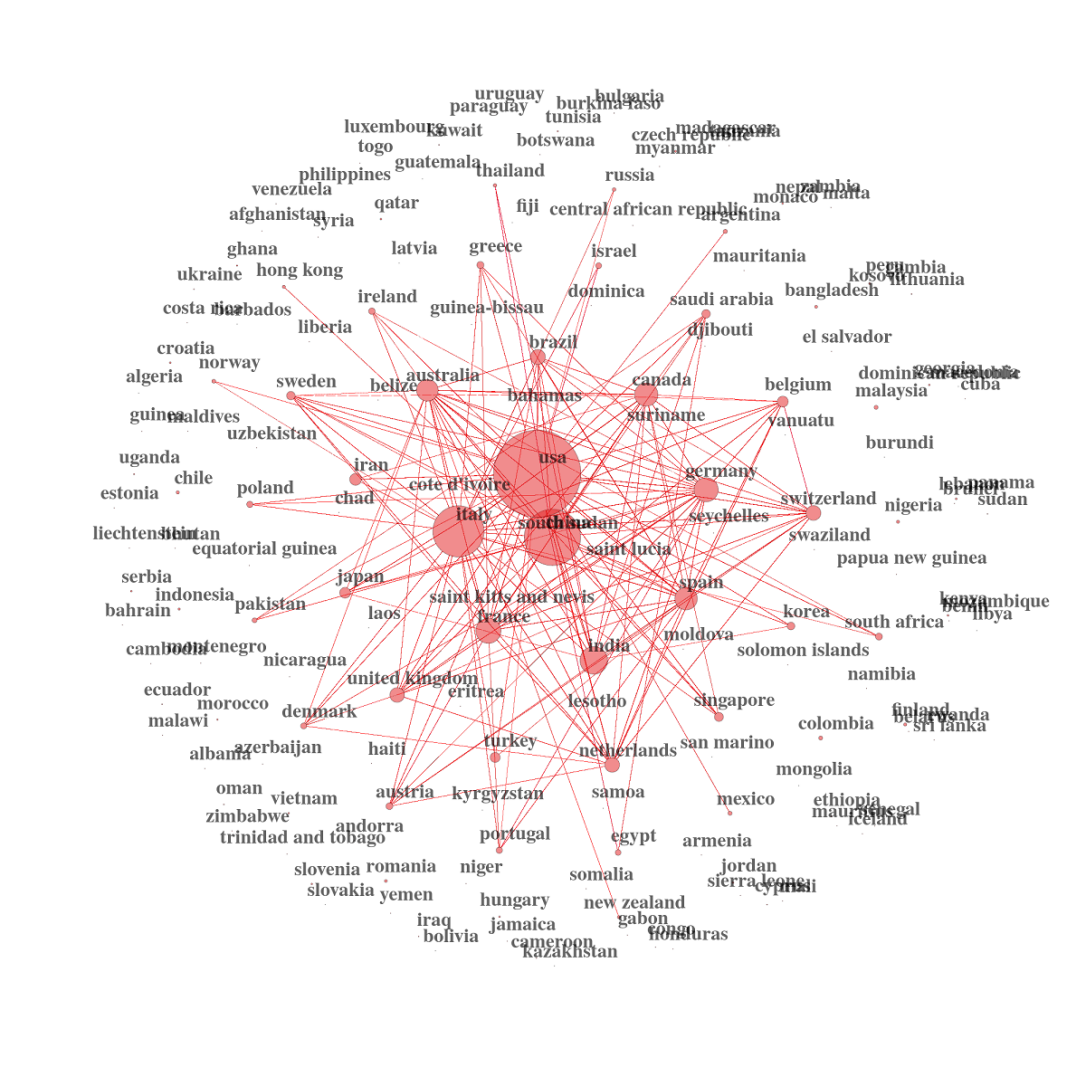
Country collaboration networks during 2020.

**Figure 19:**
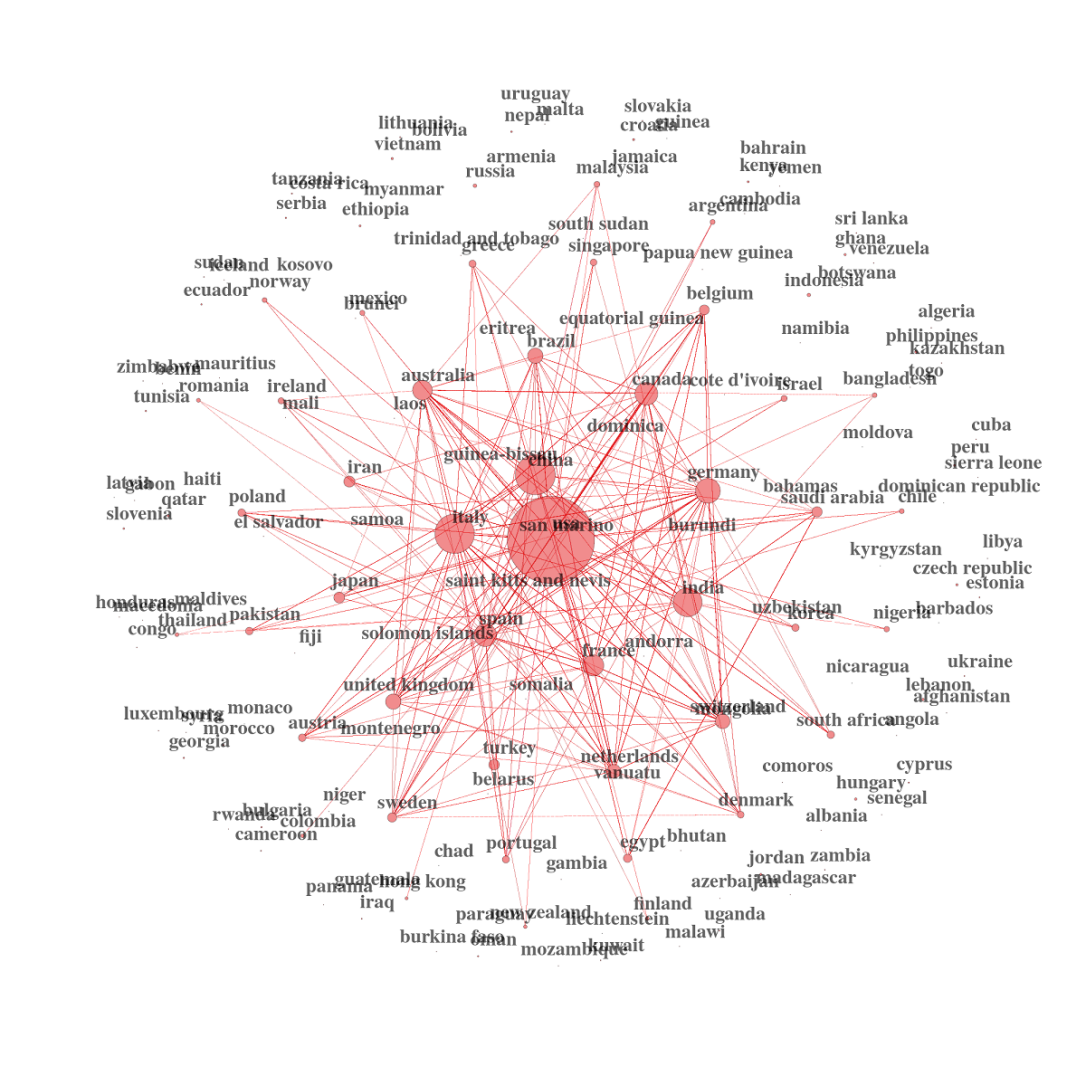
Country collaboration networks during 2021.

We can then graph the country networks using these new measures. It is, in our opinion, an excellent showcase for public health policies and decision making. It is critical information for international health organizations, research institutions, and national governments (Figures 17-19)

Considering the results mentioned above, the United States and China are at the forefront of academic production. Below, we also investigated the connections at the institutional level.

#### Co-citations of Institutional Metrics

In order to continue our social structure–oriented analysis, we made use of the collaborations that have developed among universities. We used the authors’ affiliations as relevant metadata in this case, and we created a collaboration matrix to facilitate the mapping of existing links.

**Figure 20:**
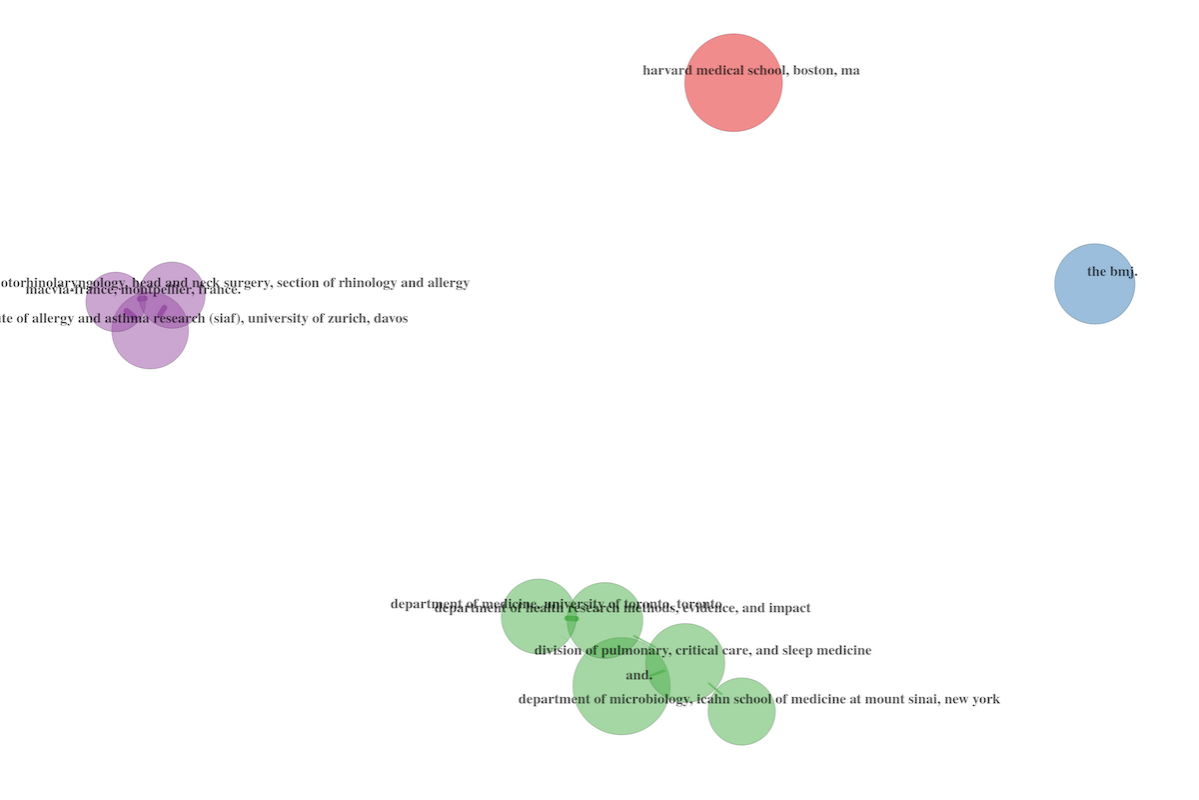
University collaboration networks during the overall period.

**Figure 21:**
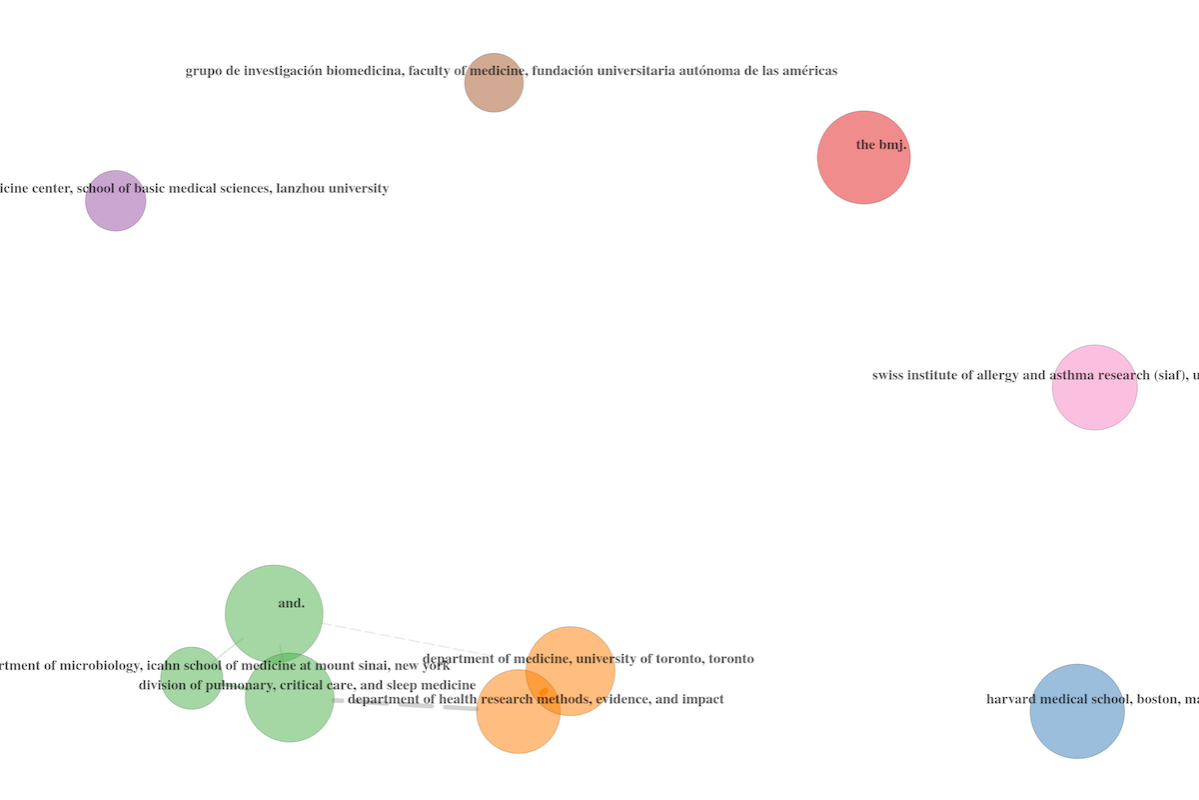
University collaboration networks during 2020.

**Figure 22:**
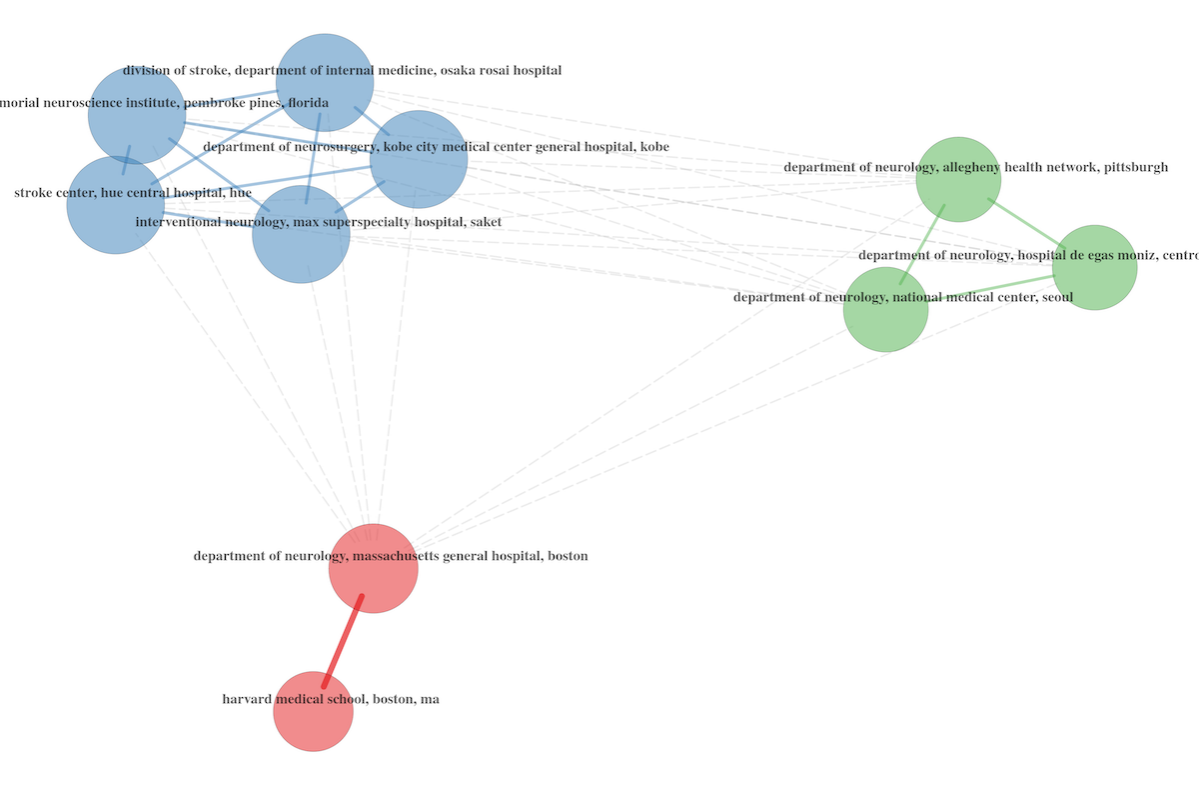
University collaboration networks during 2021.

The network of university collaborations is also worth studying for public health policy purposes (Figures 20-22), as it indicates a strong collaboration between universities within the United States, between the United States and Canada, and between the United States and the United Kingdom.

Another point worth noting is the lack of stability between 2020 and 2021, indicating that authors from various universities preferred to collaborate on topics relevant to their research rather than replicate previous collaborations. However, we only have data for 2020 and the first half of 2021 to compare, and it would require additional research to determine whether these collaborations can be sustained over time.

**Figure 23:**
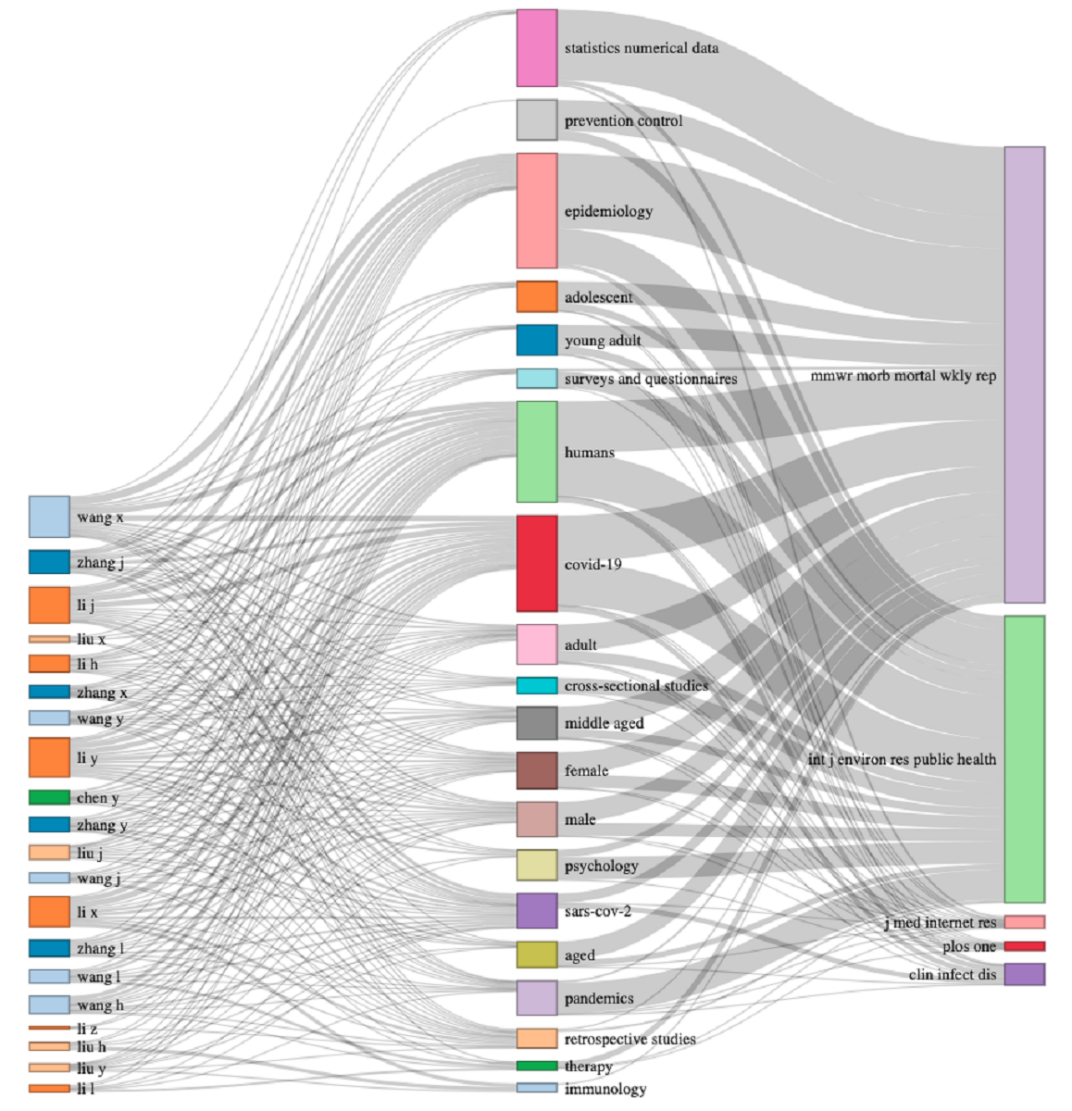
Sankey diagram of three fields representing 2020 data: authors (left), keywords (middle), and journals (right).

To summarize, Figure 23 visualizes the major components of three fields (ie, authors, keywords, and journals) and their relationships using a so-called Sankey diagram. Particularly evident in the three fields plotted in Figure 23 are the connections between the main keywords and interest in these keywords expressed by the editors of the leading journals. We can see that the majority of the journals published articles that contained the most popular keywords suggested by the authors. Currently, there are no differentiation strategies being implemented by the publishers. Figure 23 was compiled based on 25,000 documents randomly extracted, due to the computing power limits.

## Discussion

### Principal Findings

We used metadata to conduct an analysis of the global research on coronaviruses. A large portion of this analysis was carried out using data science techniques, such as NLP and structured natural language analysis. It was a time-consuming and computationally intensive task. A metadata-based approach to conducting SLRs complements more traditional methods of conducting systematic reviews of the literature. There are three axes that we used to organize the literature mapping: conceptual, intellectual, and social.

When dealing with a crisis, timing is everything. Our findings were based on the transformation of text to data and then NLP analyses of the overall global research on coronaviruses. We conducted our research in order to demonstrate what we hoped would be a proof of concept. As a result, this paper falls under the umbrella term of “action research.” It was our goal to demonstrate some metrics that can be applied to text-based documents, as well as how they could be applied to public health policies, with this proof of concept.

Our findings are, thus, essentially methodological and can demonstrate this approach’s ability to optimize global research support. In this paper, based on data science techniques, we designed some metrics, which are static in a PDF document. Now, another powerful feature is that by using the EpiBibR data package in a research pipeline based on code, we can compile those metrics in almost real time. Indeed, all those visuals can be updated on a daily basis when the package updates itself.

In terms of actionable metrics, we have discovered that most of the research was developed in 2020 and 2021, although the first article appeared in July 1949. We also learned that the United States is the leading country in terms of scientific research on this topic. China comes second, and then individual European Union members. It was also interesting to be able to identify the international collaborations between research centers, notably between the United States, Canada, and the United Kingdom. Another interesting result was being able to capture the sizes of the research fields related to the coronaviruses, such as epidemiology, pneumology, among others.

### Strengths and Limitations

Policy makers must use the most effective tools when designing public health responses in the context of the COVID-19 pandemic. Using coronaviruses as an example, this paper proposed a framework for identifying key topics and research institutions that conduct the most relevant coronavirus research.

This is especially true in the midst of what are referred to as infodemics [36]. Health policy makers may be exposed to risks associated with a lack of information, but they may also be exposed to risks associated with an overabundance of information. The quality of the information is the most important factor to consider. Indeed, one of the issues raised by WHO Director-General Tedros Ghebreyesus at the beginning of the pandemic was the “infodemic,” which is defined as the rapid spread of large volumes of information, whether true or false; the infodemic was declared on February 15, 2020 [37].

We must rely even more heavily on the contributions of the scientific community in the future. Because of advances in technology and data accessibility, policy makers today must employ the most up-to-date data science techniques in order to develop evidence-based public health policies, even more so in the COVID-19 era.

Our framework has also helped bring to light some of the limitations and biases that can be introduced into the process. These are not roadblocks, but rather concerns that a health data scientist should take into consideration. When it comes to author names, the homonymy problem serves as an excellent illustration. EDI is another aspect to consider in using those metrics. There are solutions to this problem, but they must be taken into consideration.

Another constraint is the amount of computing power required to run these machine learning routines on a large scale. National governments and international organizations, on the other hand, are not bound by this restriction in any way.

It may also be beneficial to include references from other disciplines in order to benefit from the vast number of methodologies, theories, and concepts that are available. In order to assess the spread of the disease, for example, demographers’ literature, as well as theories, would undoubtedly be relevant.

### Conclusions

This is the first time that metadata have been used to analyze global research on coronaviruses. A total of 121,231 documents have been processed, resulting in a text-as-data data set. Using machine learning and NLP techniques, we have proposed a framework for public health policy makers. This framework and its metrics have the potential to assist national governments and international organizations, such as the WHO, in identifying critical global collaborations in the fight against COVID-19. It exemplifies the utility of emerging data science techniques and new modes of thought in public health.

## Abbreviations

2019-nCoV: novel coronavirus
AI: artificial intelligence
BERT: bidirectional encoder representations from transformers
BioBERT: bidirectional encoder representations from transformers for biomedical text mining
CIRANO: Centre interuniversitaire de recherche en analyse des organisations
CORD-19: COVID-19 Open Research Dataset
EDI: equity, diversity, and inclusion
EpiBibR: epidemiology-based bibliography for R
MERS-CoV: Middle East respiratory syndrome coronavirus
NLP: natural language processing
ORCID: Open Researcher and Contributor ID
SLR: systematic literature review
SNA: social network analysis
WHO: World Health Organization
